# Evaluation and countermeasures of contracted services of Chinese family doctors from demanders’ point of view — a case study of a city

**DOI:** 10.1186/s12913-022-08891-6

**Published:** 2022-12-16

**Authors:** Songyi Liu, Wenqi Meng, Qianqian Yu, Haibo Peng, Xiaoli Jiang, Zixin Li, Wenqiang Yin, Zhongming Chen, Dongping Ma, Kui Sun

**Affiliations:** School of Management, Weifang Medical University, Baotong street No.7166, 261053 Weifang, China

**Keywords:** Primary health care, Family doctors’ contracted services, Primary care assessment tools

## Abstract

**Background:**

The “gatekeepers” for residents’ health are their family doctors. The implementation of contracted services provided by family doctors is conducive to promoting hierarchical diagnosis and treatment and achieving the objective of providing residents comprehensive and full-cycle health services. Since its implementation in 2016, the contract service system for Chinese family doctors has yielded a number of results while also highlighting a number of issues that require further investigation. Consequently, the purpose of this study is to assess the impact of family doctors’ contracted services in a Chinese city from the perspective of demanders (i.e., contracted residents), identify the weak links, and then propose optimization strategies.

**Methods:**

In this study, a city in Shandong Province, China was selected as the sample city. In January 2020, 1098 contracted residents (including 40.5% men and 59.5% women) from 18 primary medical institutions (including township health centers and community health centers) were selected for on-site investigation. Take the PCAT-AS(Adult Short) scale revised in Chinese as the research tool to understand the medical experience of contracted residents in primary medical institutions, and interview some family doctors and residents to obtain more in-depth information.

**Results:**

Among the four core dimensions of PCAT-AS, the score of Continuous was the highest (3.44 ± 0.58); The score of Coordinated was the lowest (3.08 ± 0.66); Among the three derived dimensions, the score of Family-centeredness was the highest (3.33 ± 0.65); The score of Culturally-competent was the lowest (2.93 ± 0.77). The types of contracting institutions, residents’ age, marital status, occupation, and whether chronic diseases are confirmed are the influencing factors of PCAT scores.

**Conclusion:**

The family doctors’ contracted services in the city has achieved certain results. At the same time, there are still some problems, such as difficult access to outpatient services during non-working hours, incomplete service items, an imperfect referral system, and inadequate utilization of traditional Chinese medicine services, it is recommended that the government continue to enhance and increase its investment in relevant policies and funds. Primary medical institutions should improve the compensation mechanism for family doctors and increase their work enthusiasm, improve and effectively implement the two-way referral system, gradually form an orderly hierarchical pattern of medical treatment, provide diversified health services in accordance with their own service capacity and the actual needs of residents, and improve the utilization rate of traditional Chinese medicine services in primary medical institutions.

**Supplementary Information:**

The online version contains supplementary material available at 10.1186/s12913-022-08891-6.

## Introduction

“Primary level emphasis” is one of China’s health work policies. Primary medical institutions represent the lowest level of the health service system and the most fundamental component of China’s medical and healthcare system [1]. Primary medical institutions provide medical and health services, such as basic public health, basic medical treatment, contracted family doctor services, preventive healthcare, and planned immunizations, which play an essential role in preserving the health of residents [2]. Numerous studies have demonstrated that the most prevalent and frequently occurring diseases can be identified and treated in primary healthcare facilities, which also contributes to the development of an appropriate diagnosis and treatment hierarchy and the enhancement of the effectiveness of the medical and healthcare systems [3–6].

However, by 2019, the proportion of primary medical institutions in China has reached 94.7%, but the proportion of patients receiving treatment is only 52.0% [7]. There hasn’t been much of a change in the “wartime state” of large hospitals and the “few people” of small hospitals [8]. In 2016, seven ministries and commissions, including the Medical Reform Office of the State Council, jointly issued *guidance on promoting contracted services of family doctors*, and contracted services of family doctors were implemented nationwide. The goals of these initiatives were to change the mode of primary medical and health services, strengthen the network function of primary medical and health services, and better protect the public’s health. Family physicians are primarily responsible for providing contracted services to residents, including primary medical care, public health, and health management. Basic medical services include the diagnosis and treatment of common and frequently occurring diseases with traditional Chinese and Western medicines, rational drug use, medical path guidance, referral appointments, and so forth. The public health services encompass the national basic public health services as well as other specified public health services. Health management services are primarily geared toward the health status and requirements of residents and develop a variety of personalized contracted services, such as health assessment, rehabilitation guidance, family sickbeds, family nursing, traditional Chinese medicine preventative treatment services, remote health monitoring, etc. At this time, the following individuals work primarily as family doctors: first, registered general practitioners in grass-roots medical and healthcare institutions (including assistant general practitioners and general practitioners of traditional Chinese medicines), followed by qualified doctors in township hospitals and rural doctors, and finally, qualified doctors in public hospitals and retired clinicians with intermediate or higher titles, particularly physicians in internal medicine, gynecology, pediatrics, and traditional Chinese medicine. Similarly, qualified non-governmental medical and health institutions (such as private clinics) are encouraged to provide contracted services and are subject to the same collection and payment policies. With the development of a talented team of general practitioners in the future, a contract signing service team with general practitioners at its core will emerge [9]. Residents who voluntarily choose a family doctor team to sign a service agreement specifying the content, method, and duration of the contracted services, as well as the responsibilities, rights, and obligations of both parties, are considered contracted residents. Residents can enjoy the services provided by the family doctor once they establish a contractual relationship with the doctor and become the primary beneficiaries of this policy. To enhance the family doctor system and better support the implementation and quality improvement of contracted services provided by family doctors, it is, therefore, crucial to comprehend the experience of contracted residents at the primary healthcare facilities [10].

The quality of primary care provided by family doctors in various countries and regions has been evaluated in previous studies from the perspective of the connotation of basic medical services, such as public health, preventive healthcare, chronic disease management, and reproductive health for women and children [11–14]. Different researchers have different conceptions of what basic medical services entail, and the evaluation criteria vary. However, the evaluation focuses more on the measurement indicators for preventive services and chronic disease management. To assess the impact of family doctor services from the standpoint of patient perception and patient experience, some scholars chose or created pertinent indicators [15–18]. The quality evaluation from the patient’s point of view obtains the majority of its measurement indicators via the Delphi method and its data via a questionnaire survey. The research methodologies and data collection are comparable. From the standpoint of family doctor service capability, some other researchers assessed the fundamental medical care, preventive healthcare, health management, and other service capabilities that family doctors should possess [19–21]. According to previous research, there is still a dearth of information regarding the achievement of the objectives of contracted services of family doctors, and the evaluation of contracted residents based on whether they are first diagnosed at the primary medical institutions and whether they have obtained comprehensive, continuous, and coordinated basic medical services is a crucial criterion for the achievement of the goals of family doctors’ contracted services, which corresponds to the fact that the family doctors’ contracted service in China is still in the exploratory phase, which served as the foundation for the selection of this study’s evaluation perspective.

Based on this, this study employs revised Chinese primary care assessment tools, investigates the experience and influencing factors of contracted residents in contracted institutions, and then proposes countermeasures to improve the quality of contracted services provided by family doctors. In China, some scholars applied the PCAT scale to carry out relevant research. Kuang Li, Du Juan, etc. evaluated the reliability and validity of the application of the PCAT in China and concluded that it has some applicability in China, but that it must be revised in light of China’s social culture and medical and healthcare systems [22–25]. Zhang Jindan and Lin yingyu used PCAT-AS(Adult Short) to evaluate the quality of primary healthcare services from the perspective of patients with chronic diseases and discovered that patients’ recognition of the quality of community healthcare services has a positive effect on the efficacy of hypertension management [26, 27]. Yuan Shasha and others used PCAT-AE(Adult Expanded) to examine the differences in the quality of primary healthcare under the vertical integration mode. The results indicated that the initial diagnosis of primary healthcare institutions under the vertical integration mode was more accurate, but the continuity and three derived dimensions must be strengthened further [28]. Liang Yuan and others utilized PCAT-AE to evaluate the degree of realization of general practice characteristics under various general practice modes and concluded that the functional services of general practice should be continuously enhanced in accordance with the regional conditions [29]. Liu Xiao and others utilized PCAT-AE to determine if there are differences in the service quality of community healthcare service institutions held by hospitals of varying levels [30]. Wang Wenhua, Hao Wenli and others used PCAT-AE(or PCAT-AS) to assess the quality of primary healthcare services based on patient experience [31–34]. Using PCAT, Feng Shanshan and Lina Li assessed the effect of family doctors’ contracted services on the quality of primary healthcare [35, 36]. In previous studies, first, a portion of the research time was relatively lengthy, and some PCAT scale items are no longer applicable to the present circumstance. Second, the majority of studies that investigated the service quality of primary medical institutions did not include the contracted service provided by family physicians. Third, in the study evaluating the impact of family doctors’ contracted services on the quality of primary healthcare using the PCAT, the scale was not revised in conjunction with family doctors’ contracted services’ policy context. Based on the current economic and social development in China and the health policy context of the new era, combined with the policy objectives of family doctors’ contracted services, this study revised the scale in Chinese to make it more suitable for the current economic and policy context, and the measurement elements are also more consistent with the objectives of Chinese family doctors’ contracted services, which is conducive to enhancing the credibility of the research.

## Method

All methods were performed in accordance with the relevant guidelines and regulations.

To ensure that the respondents’ privacy is fully protected, we informed them of the purpose of the study and the sample selection criteria prior to conducting the study, explained that the data collected will only be used for academic research, and obtained signed informed consent from each respondent. The principles of complete voluntariness and anonymity were the cornerstones of data collection.

### Study area and study population

This study used stratified random sampling to select the research objects. Three counties(cities, districts) were selected at random from a city in Shandong Province based on their level of economic and social development, and three township health centers and three community health service centers were selected from each county (city, district). In China, township health centers are comprehensive institutions established by counties or townships for the administration and prevention of medical conditions. They are an essential component of three-tiered medical networks in rural areas. They are responsible for handling important tasks related to medical treatment and healthcare. They are a significant factor in directly addressing the challenges and costs associated with seeing a doctor in rural areas. The purpose of the community health service centers is to address the community’s most pressing health issues and to meet its needs for basic health services, such as disease prevention, medical treatment, healthcare, and rehabilitation. It is an efficient, cost-effective, convenient, all-encompassing, and continuous primary care facility that integrates health education and family planning technical services. Both public welfare and comprehensive primary medical institutions diagnose and treat common and recurrent diseases, in addition to providing basic public health services, health management, and other operational responsibilities. Community health service centers primarily serve urban community residents, whereas township health centers primarily serve township residents. On the basis of the selected primary medical institutions, 60 contracted residents were selected at random from each institution’s contract registration book in order to conduct an investigation. The included objects were all contracted residents who could independently complete the questionnaire and articulate their thoughts clearly. A total of 1080 residents were investigated from 18 township health centers and community health service centers in three counties (cities, districts). The sample size for this survey was carefully chosen based on the need for 5–10 times the number of questionnaire items [37], the stability of the results, and other factors. Family doctors assisted the investigator in the household investigation. To ensure the quality of the investigation, all of the investigators were properly trained prior to the investigation. In the survey, an investigator was primarily responsible for evaluating the questionnaire’s quality. If there were issues, respondents were required to supplement and improve the information on-site. Following the survey, a second investigator reorganized and reviewed the questionnaire, removing the invalid ones. In addition to the quantitative questionnaire survey, some family doctors and contracted residents were selected at random for face-to-face interviews in accordance with the principle of information saturation.

### Evaluation tool of effect of family doctors’ contracted services

**Fig. 1:**
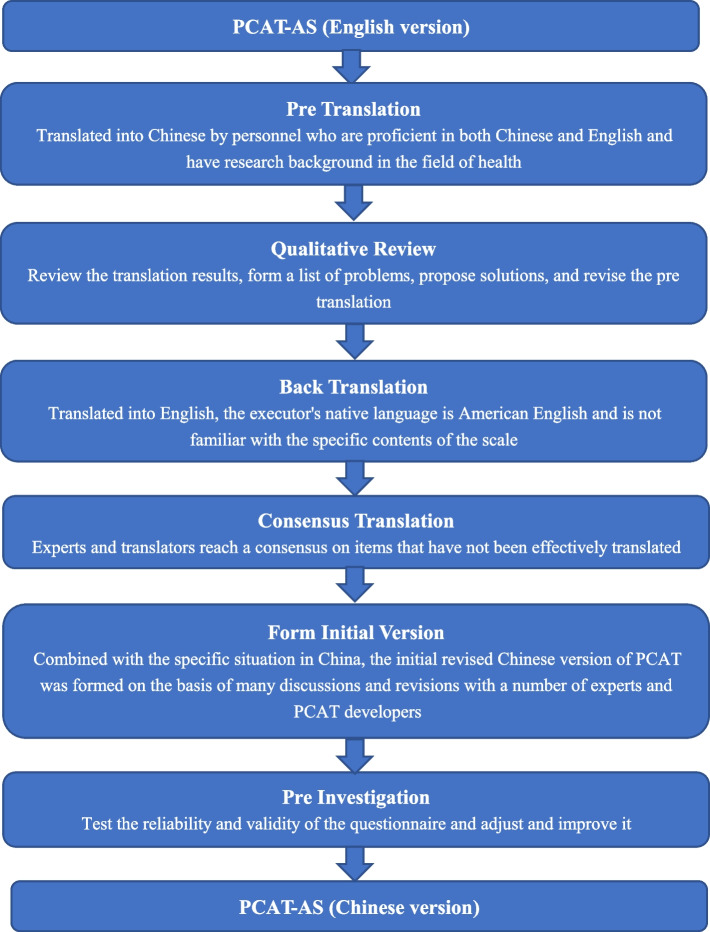
PCAT-AS translation program

PCAT demand side version is a measurement scale proposed by WHO to assess the standard of healthcare services offered by primary healthcare facilities based on the features of fundamental medical services [38, 39]. The measurement dimensions of the PCAT, such as initial contact, continuous, coordinated, and comprehensive, align with the contracted service goals of family doctors implemented in China. Therefore, with permission from the PCAT Research Institute at Johns Hopkins University, this study used the scale as a reference, consulted experts in relevant fields, combined the policy background and service objectives of contracted services of family doctors in China, revised the adult short version of PCAT (PCAT-AS) in Chinese according to the translation procedure of the scale, verified its reliability, validity, and applicability through pre investigation, improved the survey tools, revised them in accordance with the revision principles corresponding to China’s actual situation, after pre translation, qualitative evaluation, back translation, consensus translation, initial version formation, pre investigation, and other processes, and formulated the final questionnaire (Fig. 1).

### Pre translation

Two researchers, whose mother tongue is Chinese but speak English fluently and have a health management background with a work experience of at least 10 years, independently translated the original English questionnaire. Both translators are members of this research group and are acquainted with the pertinent PCAT theories and concepts. On the one hand, they could ensure that the Chinese translation of the original English questionnaire was as accurate as possible. On the other hand, they could ensure semantic consistency between the Chinese version and the original English version of the questionnaire from the perspective of English majors. After the translation was completed, the two translated versions were compared and analyzed, the items with differences were discussed and summarized, and the items that remained unresolved after discussion were added to the list of issues for further discussion and revision.

### Qualitative review

Using the pre-translation-generated list of questions, the Delphi method was used to select three health management experts with extensive experience. On the basis of a thorough consideration of China’s historical and cultural background, language expression habits and usages, and the feasibility of future field research, the problem items that did not reach an agreement were modified accordingly, the reliability and validity of the questionnaire’s content were evaluated, and the revised Chinese translation of PCAT was determined.

### Back translation

The Chinese version of the initial draft of the PCAT-AS was translated into English by two retransmitters whose native language is English, who are fluent in Chinese, and who have never seen the original English version of the PCAT questionnaire. After the back translation version was developed, it was compared and modified with the original PCAT-AS English version questionnaire in order to bring the back translation version closer to the English version in terms of language description and expression.

### Consensus translation

On the basis of the back translation version, all experts and translators who participated in the pre-translation were gathered, qualitative evaluation and back translation were performed before and after, their work was reasonably divided, consensus translation of the back translation version was performed again according to the forward and reverse translation order, and effective discussion and revision of the items that were not effectively translated were conducted. It was also ensured that the contents of each section and item were reasonably appropriate for China’s historical, cultural, and linguistic traditions. It establishes a strong foundation for the early development of the updated PCAT-AS in Chinese that is appropriate for the implementation of family doctor contract services in China.

### Form initial version

The initial version of the Chinese revision of PCAT-AS, which primarily consists of two aspects, was created based on the particular circumstances in China, on the basis of numerous discussions and revisions with PCAT developers and numerous experts involved in the implementation of policy, management, and academic research of primary medical and health undertakings: first, the options for responding to the questionnaire were revised. The five categories of Chinese descriptions for the questionnaire answer choices, particularly the “not sure/don’t remember” option, have been translated in a variety of ways by prior researchers. The second step was to revise the Chinese version of the questionnaire’s specific content. For example, in accordance with the study’s objectives, we replaced all instances of “PCP (primary care provider)” in the title with “family doctor.“ In the dimension of first contact access, for the two questions C4 and C5, due to the development of modern communication technology, the communication mode between doctors and patients has changed, so the description of the consultation mode in the above three questions has been changed from “telephone consultation” to “phone, Wechat, email, and other methods for consultation”. In terms of cultural competence, combined with the actual situation of services provided by primary medical institutions in China, K2 “Would you recommend your PCP to someone who does not speak English well?“ and K3 “Would you recommend your PCP to someone who uses folk medicines, such as cows or Homemade medicines, or has special beliefs about healthcare?“ were revised as K2 “Would you recommend traditional Chinese medicines in your contracted institution to a friend or relative?“ and K3 “Would you recommend preventive care at your contracted institution to a friend or relative?“.

### Pre investigation

We conducted a pre survey on 496 people in a city to verify the reliability and validity of the revised Chinese PCAT-AS. The Cronbach α of the seven dimensions of the scale are 0.808, 0.862, 0.892, 0.961, 0.886, 0.948 and 0.890 respectively, which are greater than 0.8 and have good reliability. Confirmatory factor analysis (CFA) was conducted on all dimensions of the scale, and the results showed that the χ2/df、GFI、NFI、CFI and RMSEA values reached the adaptation standard and the model fit well (Table 1).

**Table 1:** Model fitting of each dimension of PCAT-AS

Dimensions	*χ*^*2*^/*df*	*GFI*	*NFI*	*CFI*	*RMSEA*
First contact	4.902	0.993	0.995	0.996	0.032
Continuous	4.805	0.994	0.997	0.997	0.032
Coordinated	4.790	0.980	0.983	0.986	0.045
Comprehensiveness	4.936	0.984	0.990	0.992	0.032

Since the dimensions of "Family-centered" " Community-oriented " and " Culturally-competent" are three items, the parameters and data moments to be estimated are 6 and the degree of freedom is 0, so the above dimensions only need the factor load to meet the standard. χ 2 / df < 5 is suitable and < 3 is good; GFI value > 0.9 is suitable; NFI value > 0.9 is suitable; CFI value > 0.9 is suitable; RMSEA value < 0.05 is excellent 0.08 is good

The revised scale includes four core dimensions: First contact, Continuous (ongoing), Coordinated and Comprehensive, as well as three derived dimensions: Family-centeredness, Community-oriented and Culturally-competent. The specific connotations are as follows: First contact care means that care is first sought from the primary care provider when a new health or medical need arises, it includes two sub dimensions: “utilization” and “access"。Continuous (ongoing) care refers to the longitudinal use of a regular source of care over time, regardless of the presence or absence of disease or injury. Coordinated care is the linking of health care visits and services so that patients receive appropriate care for all their health problems, physical as well as mental, it includes two sub dimensions: “referral” and “information system"。Comprehensive care refers to the availability of a wide range of services in primary care and their appropriate provision across the entire spectrum of types of needs for all but the most uncommon problems in the population by a primary care provider, It includes two sub dimensions: “service provision” and “frequency of mentioning health problems”. Family-centeredness care recognizes that the family is a major participant in the assessment and treatment of a patient. Community-oriented care refers to care that is delivered in the context of the community. Culturally-competent care refers to care that honors and respects the beliefs, interpersonal styles, attitudes, and behaviors of people as they influence health.

The questionnaire was scored in accordance with the *MANUAL for the PRIMARY CARE ASSESSMENT TOOLS*, compiled by Barbara starfield and Leiyu Shi of Johns Hopkins University. Likert 4 subscale was used for scoring. 4 points, 3 points, 2 points and 1 point will be scored respectively for the options “will”, “may”, “may not” and “will not”. If the proportion of “uncertain / unknown” selected for each dimension is higher than 50%, no score will be given. If it is lower than 50%, 0 point will be scored for the option “uncertain / unknown” in the “comprehensive” dimension, and 2 points will be scored for other dimensions. The score of each dimension is the average of the scores of all items in the dimension, and the total score of the scale is the sum of the scores of each dimension. The highest score is 28 points and the lowest score is 7 points. The higher the PCAT score, the better the treatment experience of contracted residents at contracted institutions, indicating that the family doctors’ contracted service has a greater impact.

### Statistical analysis

SPSS and Amos software were used for statistical analysis. Cronbach’s α was used to test the reliability of the Chinese Revised PCAT-AS, and confirmatory factor analysis was used to test the validity. The demographic and sociological characteristics of contracted residents are presented by descriptive statistics, i.e. n (%), the score of PCAT scale is presented by mean ± SD, the difference of scores of different demographic characteristics was analyzed by one-way ANOVA and the influencing factors are analyzed by multiple linear regression。All statistical tests were two-sided, and *P*-values < 0.05 were deemed significant.

## Results

### Basic information of respondents

Finally, a total of 1193 contracted residents were investigated, and 1098 valid questionnaires were recovered, with an effective rate of 92%. Among them, 541 residents contracted with community health centers, while 557 residents contracted with township health centers. The proportion of respondents from the two institutions was comparable. Women outnumbered men, constituting 59.5% of the population. In terms of age, the majority of respondents (48.2%) were seniors aged 60 and older, and 58.4% of the respondents had a primary school education or less. Eight hundred and thirty-two people were married, constituting 75.8% of the population. More than half (59.0%) were in-service personnel. The income of most respondents (70.1%) was less than 2000 yuan.

In terms of health status and utilization of health services, 60.0% of the respondents rated their health status as “very good” or “good”; 45.8% of the respondents had chronic diseases; 49.4% of the respondents participated in the medical insurance for urban and rural residents, and 47.4% participated in the medical insurance for urban workers (Table 2).

**Table 2:** Demographic characteristics of respondents

**Variable**	**n(%)**
**Contracted institution**	
Township Health Center	557 (50.7)
Community Health Centre	541 (49.3)
**Gender**	
Male	445 (40.5)
Female	653 (59.5)
**Age**	
Under 18	11 (1.0)
18-29	136 (12.4)
30-39	61 (5.5)
40-49	156 (14.2)
50-59	205 (18.7)
60 and over	529 (48.2)
**Education(ref: Primary school and below)**	
Primary school and below	641 (58.4)
Junior high school	233 (21.2)
High school / technical secondary school	77 (7.0)
Junior college	54 (4.9)
Bachelor degree or above	93 (8.5)
**Marital status(ref: unmarried)**	
Unmarried	141 (12.8)
Married	832 (75.8)
Divorce	5 (0.5)
Widowed	120 (10.9)
**Occupation(ref: In service personnel)**	
In service personnel	649 (59.0)
School Students	106 (9.7)
Retired personnel	37 (3.4)
On the drift	306 (27.9)
**Personal monthly income(RMB yuan)**	
Under 2000	770 (70.1)
2000-2999	112 (10.2)
3000-3999	82 (7.5)
4000-4999	51 (4.6)
5000 and above	83 (7.6)
**Self rated health status(ref: Very good)**	
Very good	219 (19.9)
Good	440 (40.1)
General	297 (27.0)
Poor	132 (12.0)
Very poor	10 (0.9)
**Is chronic disease diagnosed**	
Yes	503 (45.8)
No	595 (54.2)
**Medical insurance**	
Urban and rural residents medical insurance	542 (49.4)
Urban employee medical insurance	520 (47.4)
Business insurance	29 (2.6)

The sum of the percentages is not equal to 100% because both urban and rural residents and urban workers can participate in commercial insurance at the same time, and some residents do not participate in any insurance

### PCAT score

**Fig. 2:**
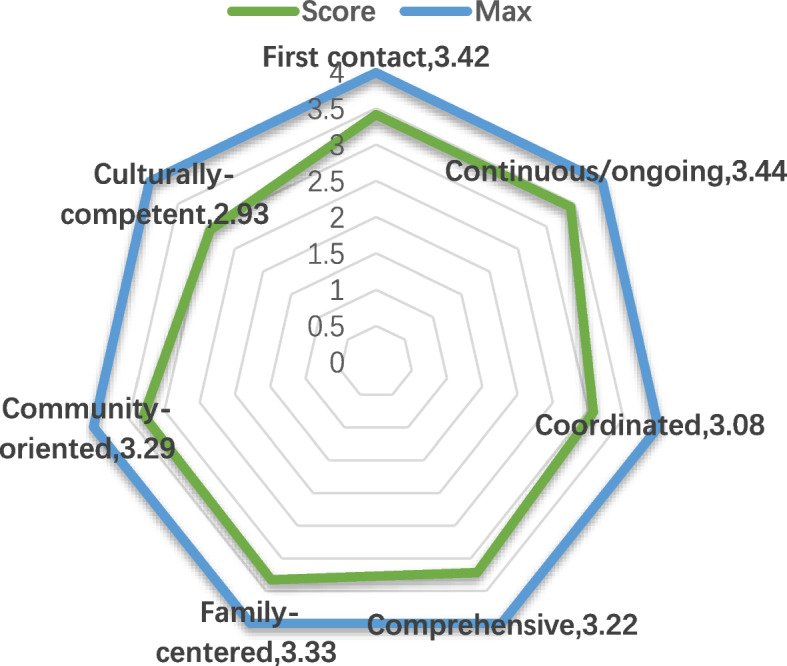
PCAT score

Overall score: the total score of PCAT in this survey is 22.72 ± 3.19, of which the First contact dimension is 3.42 ± 0.48 (the sub dimension of “service utilization” is 3.52 ± 0.61, “service accessibility” is 3.34 ± 0.52); Continuous dimension: 3.44 ± 0.58; Coordinated dimension: 3.08 ± 0.66 (the sub dimension of “referral” is 2.98 ± 0.76, “information system” is 3.13 ± 0.68); Comprehensive dimension is 3.22 ± 0.46 (the sub dimension of “service provision” is 3.29 ± 0.42, and the “frequency of mentioning health problems” is 3.17 ± 0.65); Family-centeredness dimension 3.33 ± 0.65; Community-oriented dimension: 3.29 ± 0.65; Culturally-competent dimension is 2.93 ± 0.77 (Fig. 2).

As can be seen from Fig. 2, among the four core dimensions of PCAT, the score of Continuous dimension is relatively high and the score of Coordinated dimension is relatively low; Among the three derived dimensions, the score of Family-centeredness dimension is relatively high, and the score of Culturally-competent dimension is low.

### Score of specific items

As shown in Table 3, the scores of B1 and C5 in the first contact, D1 and D9 in the continuous, G2 and H7 in the comprehensiveness and I1 in the family-centeredness are higher, more than 3.50 points; C7 in the first contact, E9, E10 and F1 in the coordination, G8, G10, H2 and H4 in the comprehensiveness, K2 and K3 in the culturally competent, the scores of the above items are low, below 3.00.

**Table 3:** Score of each item of PCAT

Items	Score
**FIRST CONTACT – UTILIZATION**	
B1When you need a regular general checkup, do you first come to your family doctor?	3.55 ± 0.71
B2When you feel unwell, will you first come to your family doctor before looking for a specialist?	3.49 ± 0.73
B3When you have to see a specialist, does your family doctor have to approve or give you a referral?	3.42 ± 0.63
**FIRST CONTACT-ACCESS**	
C3When your contracted institution is open and you get sick, would doctors or nurses from there visit you?	3.48 ± 0.73
C4When your contracted institution is open, can you get advice over the phone、WeChat、e-mail if you need it?	3.24 ± 0.91
C5When your contracted institution is closed, can you get advice over the phone、WeChat、e-mail if you need it?	3.73 ± 0.58
C7When your contracted institution is closed and you get sick during the night, would doctors or nurses from there visit you?	2.91 ± 0.85
**CONTINUOUS(ONGOING) CARE**	
D1When you go to your contracted institution, are you taken care of by your family doctor each time?	3.57 ± 0.63
D4If you have a question, can you talk to your family doctor (or team member)?	3.37 ± 0.81
D7 Does your family doctor know you very well as a person, rather than as someone with a medical problem?	3.33 ± 0.84
D9Does your family doctor know what problems are most important to you?	3.50 ± 0.69
**COORDINATION-REFERRAL**	
E8Have you ever had a visit to any kind of specialist or special service?	3.02 ± 0.86
E9Did your family doctor discuss with you different places you could have gone to get help with that problem?	2.93 ± 0.92
E10Did your family doctor help you make the appointment for that visit?	2.97 ± 0.88
E12Did your family doctor write down any information for the specialist about the reason for the visit?	3.00 ± 0.91
**COORDINATION-INFORMATION SYSTEMS**	
F1When you go to your contracted institution, do you bring any of your own medical records, such as shot records or reports of medical care you had in the past?	2.83 ± 1.09
F2Could you look at your medical record if you wanted to?	3.20 ± 0.76
F3When you go to your contracted institution, is your medical record always available?	3.36 ± 0.69
**COMPREHENSIVENESS-SERVICES AVAILABLE**	
G2Do your contracted institution provide immunizations (shots)?	3.80 ± 0.54
G6Do your contracted institution provide family planning or birth control methods?	3.45 ± 0.70
G8Do your contracted institution provide counseling for mental health problems?	2.97 ± 1.01
G10Do your contracted institution sewing up a cut that needs stitches?	2.92 ± 1.04
**COMPREHENSIVENESS-FREQUENCE OF MENTIONING HEALTH PROBLEMS**	
H1How often does your family doctor advice about healthy foods and unhealthy foods or getting enough sleep?	3.48 ± 0.76
H2How often does your family doctor mention about home safety, like getting and checking smoke detectors and storing medicines safely?	2.99 ± 0.92
H4How often does your family doctor mention about ways to handle family conflicts that may arise from time to time?	2.52 ± 1.06
H5How often does your family doctor advice about appropriate exercise for you?	3.35 ± 0.76
H7How often does your family doctor Checking on and discussing the medications you are taking?	3.52 ± 0.72
**FAMILY-CENTEREDNESS**	
I1Does your family doctor ask you about your ideas and opinions when planning treatment and care for you or a family member?	3.52 ± 0.68
I2Has your family doctor asked about illnesses or problems that might run in your family?	3.20 ± 0.90
I3Would your family doctor meet with members of your family if you thought it would be helpful?	3.27 ± 0.79
**COMMUNITY ORIENTATION**	
J1Does your family doctor ever make home visits?	3.29 ± 0.85
J2Does your family doctor know about the important health problems of your neighborhood?	3.47 ± 070
J3Does your family doctor get opinions and ideas from people that will help to provide better health care?	3.11 ± 0.79
**CULTURALLY COMPETENT**	
K1Would you recommend your contracted institution to a friend or relative?	3.16 ± 0.82
K2Would you recommend traditional Chinese medical in your contracted institution to a friend or relative?	2.69 ± 0.94
K3Would you recommend prevention care in your contracted institution to a friend or relative?	2.84 ± 0.88

### Analysis on the difference of PCAT scores of residents with different demographic characteristics

As shown in Table 4, the results of one-way ANOVA on the PCAT scores of residents with different demographic characteristics revealed statistically significant differences in the comprehensive service scores of contracted residents with different signing institutions, ages, levels of education, marital status, occupations, and personal monthly incomes, as well as chronic diseases.

**Table 4:** Analysis on the difference of PCAT scores of residents with different demographic characteristics

Variable	PCAT score	F	P
**Contracted institution**			
Township Health Center	23.56 ± 2.45	84.240	**<0.00**1
Community Health Centre	21.85 ± 3.61		
**Gender**			
Male	22.88 ± 3.28	1.988	0.159
Female	22.61 ± 3.13		
**Age**			
Under 18	22.46 ± 5.55	2.780	**<0.001**
18–29	20.37 ± 4.32		
30–39	22.55 ± 3.68		
40–49	22.88 ± 3.28		
50–59	23.08 ± 2.56		
60 and over	23.16 ± 2.63		
**Education(ref: Primary school and below)**			
Primary school and below	23.03 ± 2.69	19.903	**<0.001**
Junior high school	22.90 ± 2.98		
High school / technical secondary school	23.31 ± 3.21		
Junior college	21.81 ± 4.48		
Bachelor degree or above	20.14 ± 4.53		
**Marital status(ref: unmarried)**			
Unmarried	20.30 ± 4.47	34.557	**<0.001**
Married	23.11 ± 2.82		
Divorce	21.02 ± 1.36		
Widowed	22.90 ± 2.58		
**Occupation(ref: In service personnel)**			
In service personnel	23.35 ± 2.86	34.805	**<0.001**
School Students	20.24 ± 4.55		
Retired personnel	22.06 ± 3.08		
On the drift	22.32 ± 2.81		
**Personal monthly income(RMB yuan)**			
Under 2000	22.73 ± 3.13	2.720	**<0.001**
2000–2999	22.81 ± 3.01		
3000–3999	22.68 ± 3.52		
4000–4999	23.16 ± 2.99		
5000 and above	22.24 ± 3.73		
**Self rated health status(ref: Very good)**			
Very good	23.12 ± 3.52	1.802	0.126
Good	22.64 ± 3.11		
General	22.73 ± 3.18		
Poor	22.40 ± 2.86		
Very poor	21.25 ± 3.54		
**Is chronic disease diagnosed**			
Yes	23.01 ± 2.66	7.927	**0.005**
No	22.47 ± 3.57		
**Medical insurance**			
Urban and rural residents medical insurance	22.86 ± 3.24	0.523	0.719
Urban employee medical insurance	22.17 ± 2.58		
Business insurance	22.53 ± 2.96		

### Multiple linear regression analysis of PCAT score

Using the total PCAT score as the dependent variable, the statistically significant variables from the univariate analysis were included in the multiple linear regression analysis as independent variables.

According to the results of multiple linear regression, the contracted institution, age, marital status, occupation, and whether or not to diagnose chronic diseases influenced PCAT scores. Residents who signed up for township health centers scored higher on the PCAT than those who signed up for community health service centers. Residents who aged 18–29 scored lower on the PCAT than those of other age groups; Residents who were married and widowed scored higher on the PCAT than those who were unmarried; The PCAT score of students is lower than that of other occupations; Residents who confirmed chronic diseases scored higher on the PCAT than those without chronic diseases (Table 5).

**Table 5:** Multiple linear regression analysis of PCAT score

Variable	PCAT score	β	95%CI	*P value*
**Contracted institution**				
Township Health Center	23.56 ± 2.45			
Community Health Centre	21.85 ± 3.61	-1.181	-1.536~-0.772	**<0.001**
**Age**				
Under 18	22.46 ± 5.55	0.109	0.002 ~ 0.036	**0.030**
18–29	20.37 ± 4.32			
30–39	22.55 ± 3.68			
40–49	22.88 ± 3.28			
50–59	23.08 ± 2.56			
60 and over	23.16 ± 2.63			
**Education(ref: Primary school and below)**				
Primary school and below	23.03 ± 2.69			
Junior high school	22.90 ± 2.98	0.010	-0.411 ~ 0.572	0.747
High school / technical secondary school	23.31 ± 3.21	0.021	-0.214 ~ 0.473	0.460
Junior college	21.81 ± 4.48	-0.022	-0.719 ~ 1.365	0.543
Bachelor degree or above	20.14 ± 4.53	-0.033	-1.528 ~ 0.772	0.519
**Marital status(ref: unmarried)**				
Unmarried	20.30 ± 4.47			
Married	23.11 ± 2.82	0.226	0.564 ~ 2.807	**0.003**
Divorce	21.02 ± 1.36	-0.002	-2.930 ~ 2.717	0.941
Widowed	22.90 ± 2.58	0.151	0.259 ~ 2.837	**0.191**
**Occupation(ref: In service personnel)**				
In service personnel	23.35 ± 2.86			
School Students	20.24 ± 4.55	-0.213	-1.292 ~ 0.458	**<0.001**
Retired personnel	22.06 ± 3.08	-0.065	-2.148 ~ 0.141	**0.025**
On the drift	22.32 ± 2.81	0.006	-1.179 ~ 1.317	0.914
**Personal monthly income(RMB yuan)**				
Under 2000	22.73 ± 3.13	-0.003	0.000 ~ 0.000	0.928
2000–2999	22.81 ± 3.01			
3000–3999	22.68 ± 3.52			
4000–4999	23.16 ± 2.99			
5000 and above	22.24 ± 3.73			
**Is chronic disease diagnosed**				
Yes	23.01 ± 2.66			
No	22.47 ± 3.57	-0.444	-0.415~-0.322	**<0.001**

### Qualitative interview results

According to the principle of information saturation, 15 family doctors and 11 contracted residents were selected at random from the surveyed institutions for face-to-face interviews in order to gain a deeper understanding of the problems that exist in the contracted services of family doctors in this area. This study refers to COREQ guide [40] and reports the research object, interview outline, data collection and analysis results in a standardized and detailed manner as much as possible.

When recruiting interviewees, we also informed them of the research purpose, content, and interview procedure, including our confidentiality policy and the interviewees’ right to refuse to answer or withdraw from the interview. Each participant in an interview provided informed consent and signed an informed consent form. The interview was centered on the interview outline (Table 6), and it was followed up appropriately in conjunction with the actual situation. The average interview time was 20 ~ 30 min, and the whole process was recorded with a recording pen with the consent of the respondents. After the interview, following de privacy processing, the voice data was transcribed into words for sorting and analysis.

**Table 6:** Interview outline

	Items
1	Are there any regulations for family doctors’ non working time visits?(answered by doctors)
2	What is the current situation of referral work between superiors and subordinates?(answered by doctors)
3	Do you need surgical treatment and psychological counseling services?(answered by residents)
4	Does the institution consider adding surgical treatment and psychological counseling services?(answered by doctors)
5	Are you willing to accept traditional Chinese medicine services in the signing institution? Why? answered by residents)

### Basic information of interviewees

Among the 15 family doctors interviewed, 8 were male and 7 were female; 1 with technical secondary school education, 8 with junior college education and 6 with bachelor’s degree; The average age was 40.9 ± 7.0; The average length of service was 16.0 ± 8.7.

Among the 11 contracted residents interviewed, 4 were male and 7 were female; 10 married, 1 widowed; There are 2 people with primary school education, 6 people with junior high school education and 3 people with high school education; The average age was 62.3 ± 11.0; 6 had chronic diseases.

### Main interview results

After sorting out and summarizing the interview data of 26 respondents, 4 themes and 10 subthemes were finally summarized, including low enthusiasm of some family doctors, poor referral work, large demand for psychological counseling services but insufficient provision and the service capacity of traditional Chinese medicine needs to be strengthened. Table 7 lists the key information obtained in the interview.

**Table 7:** Themes and subthemes

Themes	Subthemes
Some family doctors have low enthusiasm	①Less pay for overtime work during non working hours ②Small financial support for medical insurance
The referral work was not carried out smoothly	①Residents can go to the superior hospital without issuing a referral form ②Residents who issue a referral form still cannot enjoy the preferential reimbursement ratio
Psychological counseling services are in great demand but insufficient	① Psychological pressure caused by economic problems ②Patients with chronic diseases accompanied by psychological depression ③Family doctors have no energy to carry out extra work ④the primary level is lack of psychological practitioners and professional facilities and equipment
The service capacity of traditional Chinese medicine needs to be strengthened	①Lack of talents in traditional Chinese Medicine ②The service quality of traditional Chinese medicine is not high

Some family doctors have low enthusiasm. When understanding the situation of family doctors’ visits during non-working hours, some family doctors stated that there is essentially no subsidy for doctors’ visits during non-working hours; consequently, their enthusiasm is low. Some physicians elaborated on the reasons for their lack of enthusiasm. At present, the majority of the funds for contracted services come from the basic public health service funds, the medical insurance support is negligible, the signing fee is not implemented, and family physicians have a low sense of gain (Table 8).

**Table 8:** Interview content quotation

Original interview records
“At present, there is no mandatory requirement for non working time visits, and there is no additional subsidy for this work, so we generally do not visit after work, unless there are special patients who make an appointment in advance.“
“Now the workload is relatively large, and the income is not very high. The signing service funds mainly come from basic public health services. Originally, there was a subsidy of 60 yuan for signing a person, but the medical insurance bureau did not implement this fund, and the incentive mechanism and financial compensation need to be strengthened.“

The referral work was not executed efficiently. At present, although a two-way referral mechanism has been established between primary medical institutions and superior hospitals, it has not been effectively implemented. Patients who have not been issued a referral form at the primary level can still seek treatment at the superior hospital, and there is no preferential reimbursement ratio for patients who have a referral form to see a doctor at the superior hospital (Table 9).

**Table 9:** Interview content quotation

Original interview records
“ We can issue a referral form, but it’s not much. They are used to self referral,. because it’s no different that we refer them to the hospital and they go to the hospital by themselves “
“After our referral, there is no green channel to queue less or reimburse more for one package. These are not available. It is a treatment to go to the superior hospital with patients themselves.“

Psychological counseling services are in high demand but insufficient. When asked about relatively insufficient services in the current institutions, a number of residents cited the need for mental health counseling. The interviewees discovered that their psychological problems are primarily caused by economic stress, and patients with chronic diseases experience some abnormal emotional changes as a result of the impact of diseases on their physical status. At present, family physicians report that the current workload is heavy and that there is no time to provide additional services. Psychological counseling services require professional skills, equipment, etc., and grass-roots medical institutions are unable to provide this service (Table 10).

**Table 10:** Interview content quotation

Original interview records
“The epidemic situation is repeated, our work is unstable, and there are old people and children at home, so we do have some psychological pressure. Sometimes we hope that the grass-roots hospitals can have psychological counseling services” (resident)
“I have been suffering from hypertension for many years, because I have been taking medicine for this disease. Sometimes I feel irritable and can’t lift my spirits. The doctor also told me to pay more attention to mental health at ordinary times” (resident)
“Now our workload of basic medical services and basic public health services has been particularly heavy, and we really have no energy to do anything else” (doctor)
“How can this work be carried out at the primary level without professional psychological counseling talents, and without psychological counseling rooms, therapeutic instruments, etc.?“ (doctor)

Traditional Chinese medicine requires a strengthening of its service capacity. At present, the majority of primary care medical institutions offer traditional Chinese medicine services, but there are few specialists in traditional Chinese medicine diagnosis and treatment. Additionally, the national medical hall must continue to expand, and the service capability of grass-roots traditional Chinese medicine must be enhanced (Table 11).

**Table 11:** Interview content quotation

Original interview records
“There are no doctors specializing in traditional Chinese medicine here in primary hospitals, let alone any famous traditional Chinese medicine doctors. I personally recognize traditional Chinese medicine. When I need to see traditional Chinese medicine, I still go to a special traditional Chinese medicine hospital.“
“At present, there is still much room for improvement in the construction of the national medical hall, such as the equipment of traditional Chinese medicine diagnosis and treatment, the appropriate technology of traditional Chinese medicine, and so on. I hope it can be supported by finance and policy.

## Discussion

### Chinese revision of PCAT

In the past, although some Chinese scholars have revised the PCAT scale in Chinese, verified its good reliability and validity, and applied it to primary healthcare-related research, with the passage of time, the service capacity of China’s primary medical institutions has been developed to a certain extent, and relevant health policies have also been revised and reformed. Concurrently, China began implementing family doctors’ contracted services in 2016. Consequently, some items in the original Chinese version of the PCAT scale are unsuitable for measuring the efficacy of Chinese family doctors’ contracted services. Therefore, with the authorization of Johns Hopkins University, the PCAT development institution, this study consulted experts in relevant fields, combined the policy background and service objectives of the contracted services of family doctors in China, revised the PCAT-AS in Chinese again according to the scale translation procedure, improved the research tools, and tested the reliability and validity of the revised PCAT-AS in Chinese through pre investigation. After verification, its reliability and validity were found to be satisfactory, and it is suitable for the specific practice of contract service implementation by domestic family doctors.

### Effect evaluation of family doctors’ contracted services based on PCAT

The PCAT score of the city is 22.72. Compared with similar domestic studies, the score is basically the same as that in Shanxi, Jilin and other central and northern provinces [32, 33], but relatively lower than that in Guangdong, Shanghai and other southeast coastal developed provinces (municipalities directly under the central government) [26, 34]. The similarity is that the scores of the above different regions in the coordination referral dimension are low. Compared with a study in Spain, the region has higher scores in the first visit and continuity dimensions, and lower scores in the coordination and comprehensive dimensions [41], but the overall score is higher than a study in the coastal provinces of Canada [42]. However, due to the great differences in the economic and policy backgrounds of primary medical institutions in the above areas, this comparison should be treated with caution.

This study demonstrates that the city has achieved specific achievements with respect to the contractual services of family physicians. Several local and international research demonstrate that the introduction of contractual services for family physicians increases the quality of primary healthcare [36, 37, 43–48].

In the first contact dimension, contracted residents are willing to visit their family doctor first when they need to see a doctor, and they are able to consult by telephone during nonworking hours, indicating that the service capacity and quality of primary medical institutions have been developed and recognized to a certain extent by residents. In the continuous dimension, the family doctor is responsible for each visit, ensuring that he or she has a thorough awareness of the patient’s condition so that he or she can alter the diagnosis and treatment plan based on past treatment outcomes and support the patient’s rehabilitation. In the dimension of comprehensiveness service provision, the contracted institutions provided good vaccination services, which is closely related to the *National Standard for Basic Public Health Services (Third Edition)* issued by the former National Health and Family Planning Commission. The document stipulates that primary medical institutions must offer 14 fundamental public health services, including vaccination, maternity health management, and traditional Chinese medicine health management. Consequently, China’s primary medical institutions can essentially guarantee the provision of such public health initiatives. Since Chinese family doctors are responsible for providing residents with basic medical care, basic public health, health management, and other services, it is their duty to inquire about residents’ drug usage. In the family-centeredness dimension, family doctors consult patients and their families when formulating diagnosis and treatment plans, which reflects the respect for patients’ autonomy and participation in treatment.

This investigation also uncovered issues with the execution of contractual services provided by family physicians in this region. In the dimension of first contact, it is difficult for residents to get outpatient services during non-working hours. According to the interview with the relevant person in charge, there is no clear rule in China regarding the outpatient services of primary medical facilities. Instead of arranging personnel to be on duty during working hours, primary medical institutions typically provide outpatient care for residents with common and frequently occurring ailments. Visits are made only when residents are extremely ill, and the process takes time and necessitates the residents’ names, ages, service content, and other information. Therefore, it is suggested that institutions utilize the mobile platform to give residents mobile booking registration methods while keeping conventional means such as telephone booking for the elderly and adhering to a blend of traditional services and intelligent innovation. In addition, we should enhance the remuneration mechanism for non-working time visits, safeguard the interests of visiting physicians, and increase the excitement of physicians during non-working time visits. Simultaneously, in light of the problem of limited medical insurance support expressed by family doctors in the interview, we should strengthen the incentive mechanism, raise medical insurance fund support for signing fees, and improve family doctors’ sense of acquisition.

The coordination dimension illustrates the challenges that arise with medical referrals in China. Although basic medical institutions have essentially established a two-way referral relationship with at least one higher-level hospital, Chinese residents can “select their own medicine,“ according to the relevant person in charge. Even if the basic family doctor does not contact the higher-level hospital for referral, residents can still independently seek treatment at the higher-level hospital, and the preferential reimbursement ratio for contracted residents who issue the referral form to see a doctor at the higher-level hospital has not been fully implemented. At the same time, economic interests, the task index of higher-level hospitals to undertake the utilization rate of beds, and the belief of some residents that there may be risks in the referral of incomplete recovery to primary medical institutions create obstacles in the downward referral from higher-level hospitals, creating a dilemma of “easy upward referral and difficult downward referral.“ Therefore, we should improve and effectively implement the two-way referral system, quantify and grade the referral indicators, clarify what types of patient situations and diseases can be transferred up or down [49], rationally coordinate the distribution of referral benefits between superior and subordinate hospitals, unblock the two-way referral channels, increase the proportion of residents receiving reimbursement after a transfer, encourage patients with stable conditions and convalescent periods to transfer to the grass-roots level, and develop a hierarchical and ordered medical treatment pattern consisting of “first diagnosis at the grass-roots level, two-way referral, acute and slow treatment, and upper and lower linkage” [50].

Since the *National Standard for Basic Public Health Services (Third Edition)* has not yet included surgical treatment, psychological counseling, and other services, there are no statutory requirements for such projects, thus the provision is somewhat lacking with regard to comprehensiveness. On the other hand, according to the relevant person in charge, the ability of some institutions to provide additional services is limited due to a shortage of funding, equipment, and personnel; However, the interview revealed that a number of residents had a need for these services, particularly psychological counseling services. In addition, family physicians consider that dealing with family conflicts and other issues is not within their scope of competence; hence, the provision of this service is inadequate; yet, family conflicts may also impair the physical and mental health of residents. It is suggested that contracting institutions, on the premise of ensuring basic public health services required by the policy, provide diversified health services and enrich the service content based on their own service ability and the actual needs of residents, such as establishing comfortable and private interview rooms and emotional catharsis rooms, introducing psychological counselors, and conducting timely counseling and mental health knowledge popularization for major psychological problems.

In the dimension of being culturally competent, residents’ recommendations of traditional Chinese medicine (TCM) services at primary medical institutions are low. TCM is the traditional Chinese medicine, which plays a vital role in sustaining the health of the Chinese people. To satisfy the needs of patients seeking TCM services, most primary medical institutes in China at present have established a “national medical hall”. However, we discovered that the majority of people continue to get Western medicine treatment at primary care facilities. Residents claim that if they require TCM services, they will visit a specialized hospital. Therefore, we should strengthen policy support for the “Guoyitang” project, promote the construction of its talented team and service capacity, so as to increase the utilization rate of TCM services in primary institutions, maximize the characteristics and advantages of TCM, and ensure the healthy development of the “Guoyitang” project construction [51].

### Different demographic characteristics affect the PCAT score

The PCAT score is affected by demographic factors such as signing agency, age, and marital status, according to this study. Residents of contracted township healthcare facilities have higher PCAT scores than people of contracted community healthcare centers. This may be due to differences in health awareness, health literacy, and medical service requirements between urban and rural residents [52]. On the one hand, the demand for medical care among rural residents is lower than that of urban residents, making it psychologically easier to meet. Furthermore, rural communities benefit from the proximity of township health centers. Villagers can establish a good doctor–patient relationship with medical personnel after receiving extensive care in health centers or village clinics, allowing contracted residents of township health centers to have a better treatment experience. On the other hand, the medical treatment behavior of urban residents is generally “higher”, and they are more willing to go to large hospitals with high accessibility and rich medical resources, whereas the phenomenon of overcrowding in large hospitals and fewer patients in community health service centers causes a slight deviation in urban residents’ perceptions of community health service centers [53]. PCAT scores are lower among students than among residents of other vocations. This may be due to students’ exposure to health information in the classroom and their higher expectations for primary care services. The scores of unmarried residents are lower than those of people with other marital statuses, which may be related to the fact that we discovered a substantial overlap between these individuals and those who are students. The contracted residents with confirmed chronic diseases scored higher than those without confirmed chronic diseases, showing that patients with chronic disorders acknowledged the benefit of contracted institutions on chronic disease management.

The advantage of this study is that the PCAT scale, which is consistent with the objectives of Chinese family doctors’ contracted services, is used as an investigation tool, and the Chinese revision is conducted in conjunction with the actual situation in China, which not only enhances the credibility of the study but also provides a new research perspective for the evaluation of the contracted service effect of family doctors. The limitations are as follows: First, the elderly are one of the primary categories for whom Chinese family doctors are contracted, hence the bulk of this study’s participants are elderly. Second, because this study is a cross-sectional survey and not a longitudinal study with multiple time points, it is unable to fully demonstrate the causal relationship between the implementation of family doctors’ contracted services and the improvement of the service level of primary medical institutions; thus, it cannot reflect the net effect of the implementation of family doctors’ contracted services.

## Conclusion

In conclusion, this study demonstrates that, since the adoption of the family doctors’ contract service system in the city, several primary medical institutions have actively studied and developed a contract service model that matches their specific needs and has yielded certain results. For instance, the first visit rate of contracted residents at primary medical institutions has increased, the connection between doctors and patients has been strengthened, better basic medical services and basic public health services have been made available, and the development of the medical information system is proceeding without incident. In addition, for the problems identified in the study, such as difficult access to outpatient services during non-working hours, incomplete service items, an imperfect referral system, and inadequate utilization of traditional Chinese medicine services, it is recommended that the government continue to enhance and increase its investment in relevant policies and funds. Primary medical institutions should make full use of Internet technology and innovate service mode, improve the compensation mechanism for family doctors and increase their work enthusiasm, improve and effectively implement the two-way referral system, gradually form an orderly hierarchical pattern of medical treatment, provide diversified health services in accordance with their own service capacity and the actual needs of residents, and fully exploit the characteristics and advantages of traditional Chinese medicines to improve the utilization rate of traditional Chinese medicine services in primary medical institutions.

## Supplementary Information

**Additional file 1.**

## Data Availability

The data of this manuscript comes from the questionnaire survey of contracted residents. All data generated or analysed during this study are included in this published article and its supplementary information files.
